# Possible Neuroprotective Mechanisms of Physical Exercise in Neurodegeneration

**DOI:** 10.3390/ijms21165895

**Published:** 2020-08-16

**Authors:** B. Mahalakshmi, Nancy Maurya, Shin-Da Lee, V. Bharath Kumar

**Affiliations:** 1 Institute of Research and Development, Duy Tan University, Da Nang 550000, Vietnam; b.mahalakshmibharath@duytan.edu.vn; 2 Department of Botany, Government Science College, Pandhurna, Chhindwara, Madhya Pradesh 480334, India; nm_7582@rediffmail.com; 3 Department of Physical Therapy, Asia University, Taichung 41354, Taiwan; 4 Department of Physical Therapy Graduate Institute of Rehabilitation Science, China Medical University, Taichung 40402, Taiwan; 5 Department of Medical Laboratory Science and Biotechnology, Asia University, Taichung 41354, Taiwan

**Keywords:** Alzheimer’s disease, anti-inflammation, antioxidant, Irisin, neurodegenerative diseases, Parkinson’s disease, physical exercise

## Abstract

Physical exercise (PE) improves physical performance, mental status, general health, and well-being. It does so by affecting many mechanisms at the cellular and molecular level. PE is beneficial for people suffering from neuro-degenerative diseases because it improves the production of neurotrophic factors, neurotransmitters, and hormones. PE promotes neuronal survival and neuroplasticity and also optimizes neuroendocrine and physiological responses to psychosocial and physical stress. PE sensitizes the parasympathetic nervous system (*PNS*), Autonomic Nervous System (ANS) and central nervous system (CNS) by promoting many processes such as synaptic plasticity, neurogenesis, angiogenesis, and autophagy. Overall, it carries out many protective and preventive activities such as improvements in memory, cognition, sleep and mood; growth of new blood vessels in nervous system; and the reduction of stress, anxiety, neuro-inflammation, and insulin resistance. In the present work, the protective effects of PE were overviewed. Suitable examples from the current research work in this context are also given in the article.

## 1. Introduction

Neuroprotection broadly means the prevention of neuronal cell death by intervening and inhibiting the pathogenetic process that causes cellular dysfunction and death. The concept of neuroprotection has attracted significant interest among the scientific world in the search for novel therapies that can help preserve brain tissue and improve overall outcome [1]. Aging is the most important risk factor for the majority of neurodegenerative diseases (like Alzheimer’s and Parkinson’s disease)in elderly individuals [2,3]. Alzheimer’s disease (AD) prevalence in individuals aged ≥95 years in the USA is ~50% [4] and Parkinson’s disease (PD) affects 2–3% of individuals aged ≥65 years [5]. Epidemiological studies have found that physical activity reduces the risk of AD and dementia by 45% and 28%, respectively [6].

**Figure 1:**
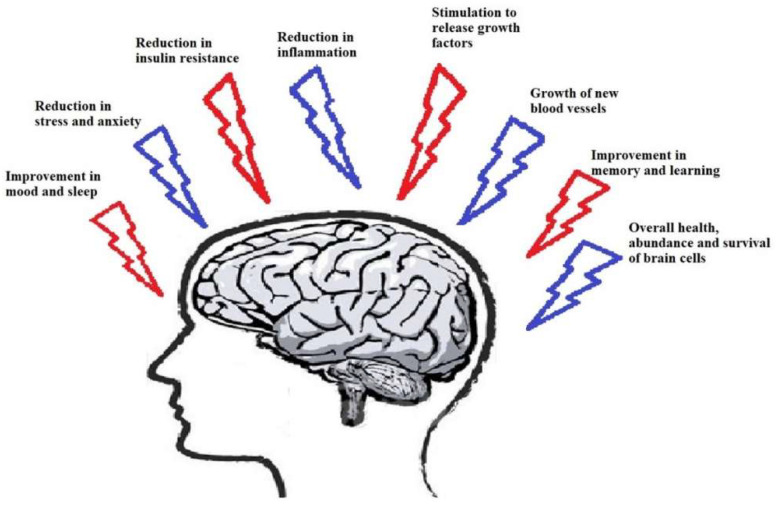
Effects of exercise on the human brain.

Based on previous studies [7,8], PE has received greater attention as a potential disease-modifying treatment approach [9]. PE has been described as a non-drug therapy against numerous diseases like neurological diseases, metabolic diseases, psychiatric diseases, and cardiovascular diseases [10]. For example, Lu et al. examined the beneficial effect of treadmill exercise upon cognitive function in a streptozotocin (STZ)-induced AD rat model.Treadmill exercise significantly inhibited neuronal apoptosis in the rat hippocampal CA1 region [11]. Tang et al. demonstrated treadmill exercise induced angiogenesis possibly by upregulating MT1-MMP expression, thereby providing protection against cerebral ischemia in rats [12]. Data from in vivo studies and human patients with neurodegeneration have proved that exercise improves cognitive performance [13,14]. With the major advances in molecular techniques, researchers have identified various molecules that are induced by PE [15] such as increased superoxide dismutase (SOD), brain-derived neurotrophic factor (BDNF), endothelial nitric oxide synthase (eNOS), insulin-like growth factor (IGF), vascular endothelial growth factor (VEGF), and nerve growth factor (NGF) and decreased harmful free radicals production in hippocampus region of brain, which are mainly involved in memory [16]. Thus, PE brings about many interrelated positive effects in the brain, which have been summarized in Figure 1. 

PE is known to slow down the process of such neurodegeneration. Regular physical activities can modulate the potential risk factors of dementia [17] and other neurodegenerative disorders like AD, PD, and others. Recently, a meta-analysis prepared evidence on the safety and efficacy of physical exercise as an additional therapeutic intervention for the quality of life, cognition, and depressive symptoms across six chronic brain disorders. These disorders were Huntington’s disease, AD, PD, multiple sclerosis, unipolar depression, and schizophrenia. This meta-analysis showed that 69% of the studies reported no complications due to exercise [18]. The study also suggested that exercising is superior to usual treatment in improving quality of life, depressive symptoms, attention, working memory, and psychomotor speed [18]. Chang et al. (2010) had suggested on the basis of their Age Gene/Environment Susceptibility Reykjavik Study in this regard that midlife physical activity may contribute toward the maintenance of cognitive function and may help delay or reduce the risk of late-life dementia [19]. Modifiable lifestyle factors such as physical activity and diet modulate common neuroplasticity substrates (neurogenesis, neurotrophic signaling, inflammation, antioxidant defense, and stress response) in the brain and hence these are considered to be important alternative therapeutic options for conditions like dementia that develop with age [20]. A study done among school children demonstrated a positive correlation between physical activity and their academic performance [21]. A meta-analysis of 29 randomized controlled trials (*n* = 2049) demonstrated that individuals doing aerobic exercise exhibited improvement in memory, attention, processing speed, and executive function [22]. 

Beneficial effects of exercise include increased blood flow from brain to the hippocampus and increases in its size in humans [23] and decreased neuro-inflammation [24]. Silverman and Deuster (2014) suggested that regular physical activity affects the following biological pathways: (i) optimization of neuroendocrine and physiological responses to psychosocial and physical stressors; (ii) acting as buffer against stress and stress-related diseases/chronic diseases; (iii) promotion of anti-inflammatory state; and (iv) enhancement of neuroplasticity and growth factor expression [25]. Not only is the functionality of the brain affected by physical activity, but the structure is also altered due to it for which there is clinical evidence. For instance, a neuroanatomical study of people between 55 to 79 years of age showed that age related reduction in the cortical tissue density of the temporal, frontal, and parietal cortices was improved significantly as a function of cardiovascular fitness [26]. An animal model-based study also supported similar findings where it was demonstrated that long-term voluntary wheel running among rats changed their spine density and also altered arborization and spine morphology [27]. The study reported that long-term voluntary running increased the density of dendritic spines in the hippocampus, granule neurons of dentate gyrus, CA1 pyramidal neurons, and in layer III pyramidal neurons of the entorhinal cortex of adult rats [24]. Upon reviewing the studies related to the neuroprotective effects of physical activity on the brain in AD using the MEDLINE database search, it was found that physical activity attenuates AD related neuropathology and brings about positive effects in hippocampus mediated cognitive function, especially when started early in the disease process; however, there is a lack of evidence in the literature to support the exact physical activity guidelines [28]. On the basis of the 38 animal and human studies that met the desired criteria in this study, it was suggested that incorporating regular physical activity in daily routines mitigates AD related symptoms, especially if adopted earlier in the disease process [25]. Another meta-analysis showed that physical activity is beneficial for patients with PD specifically in areas such as quality of life, gait, speed, balance, strength, and physical functioning [29]. Exercise effects have also been shown to decrease PD by 33% [30]. 

## 2. Physical Exercise and Neurodegenerative Disease

Lifestyle without sufficient exercise training may increase the risk of stroke, AD, and PD [31]. In older adults, aerobic exercise showed improvement in cognitive function [32]. Monteiro et al. (2015) suggested two hypotheses to explain the underlying mechanism: (a) PE reduces chronic oxidative stress along with stimulating mitochondria biogenesis and upregulation of autophagy in PD; and (b) exercise stimulates the synthesis of neuro-transmitters like dopamine and trophic factors like Glial-derived neurotrophic factor (GDNF), insulin-like growth factor-1 (IGF-1), brain derived neurotrophic factor (BDNF), and fibroblast growth factor 2 (FGF-2) [30].

PE affects many neurophysiological aspects and pathways such as autophagy, neuronal plasticity, neurogenesis, anti-oxidant defense mechanisms, and more. It also decreases neural apoptosis and neurodegeneration. PE can induce neuro-plastic changes in the human brain but with a wide inter-individual variability [17]. Regular PE is an effective autophagy inducer [33] and improves neurological function [12]. It also reduces chronic oxidative stress and promotes mitochondrial biogenesis. It also promotes the expression of neurotrophic factors like BDNF, GDNF, neurotransmitters like dopamine and hormone irisin, while downregulating Bax and neuro-inflammatory cytokines in the hippocampus [34]. Regulation of BDNF through physical exercise is a major key point as BDNF is a multifunctional molecule that has a role in neuronal plasticity, synaptic transmission and plasticity, neuronal stress resistance, differentiation and maturation of neurons, activation of other supporting molecules like NFκB, and dopamine in the neurons [15,35]. Thus, PE brings about many interrelated positive effects in the brain, which have been summarized in Figure 1.

AD is the most common form of dementia and is a major challenge for healthcare in the 21st century [36]. It is expected that in the U.S., about 15 million people (>65 years) will have AD by 2050 [37]. Since no disease-modifying treatment has been available until now, AD patients are normally treated with combined pharmacological drugs, counseling, and social care to slow down the disease progression [9,38]. Exercising is a non-pharmacological strategy that may help in protecting against cognitive decline and decrease the risk of AD [39]. PE helps stabilize and improve the cognitive function in AD patients and reduces and delays the onset of severe neuro-psychiatric symptoms like apathy, confusion, and depression [40]. Exercise has also been shown to induce anti-inflammatory effects [41] and neurotrophic factors [42]. An experimental study done in mice suggests that exercise prevented obesity-induced white matter damage by suppressing neuro-inflammation and vascular dysfunction despite significant weight gain [43]. Aerobic training significantly increases the mRNA expression of ABCA1, which may improve cognitive function by improving and preventing symptoms of AD [44]. All these findings provide treatment options for age-related neurodegenerative disorders like AD.

PD is the second most frequent age related neurodegenerative disease [45]. At the cellular level, its pathology involves dopaminergic degeneration and accumulation of cytosolic protein α-synuclein, linked with impaired autophagy-lysosome pathway (ALP) clearance [29]. Considering its therapeutic aspect, many efforts have been made using different approaches, but even after many advances in its treatment that slow down its progression and minimize locomotor impairment, its clinical management is still a challenge [46]. Recent clinical finding data showed that only high-intensity tread mill exercise training could successfully improve motor symptoms in PD patients [47]. Aerobic walking in mild to moderate PD patients was safe, well-accepted, and improved aerobic fitness, fatigue, motor function, mood, and quality of life [48]. Multi-component physical training (for eight weeks) in PD patients improved gait speed and functional status functional status [49]. In another experiment, voluntary exercise on a running wheel increased DJ-1, Hsp70, and BDNF concentrations and decreased α-synuclein aggregation in the brains of exercising mice compared to the control mice [50]. Biochemical analysis done in the same study showed that running mice had significantly higher concentrations of Hsp70, BDNF, and DJ-1 [50]. Thus, this in vivo study is strong evidence to support the notion that exercising may slow down PD progression through the prevention of abnormal protein aggregation in the brain [50]. Physical activities such as horseback riding have also been seen to improve balance and cognitive impairment in aged adults suffering PD, as described in a recent simulation study [51]. Many studies have indicated that exercise can enhance brain function and also attenuate neurodegeneration [52]. Neuroplasticity is improved by changing the synaptic structure and function in different regions of brain and also modulates multiple systems that regulate glial activation and neuro-inflammation [52]. Furthermore, exercising, in addition to carvacrol (a food additive), is also helpful in reducing rotational behavior and improves aversive memory deficit and decreases lipid peroxidation levels, along with increasing total thiol concentration in the hippocampus and/or hemi-Parkinson rats [53]. This indicates that this combination of carvacrol and treadmill exercise can be an effective therapeutic tool to treat neuro-behavioral deficits in PD patients [53]. Regular exercise also contributes to health in PD patients as it improves the ability of the patient to adapt to barriers encountered during gait, regardless of the medication state [54].

A preliminary study done on 36 PD patients reported the effects of coordination and manipulation therapy in which patients performed various activities like dry land swimming and para-spinal muscle stretching for 30 min every day for one year, while the control group did not exercise regularly. It was found that the treated group exhibited improved balance, mobility disorder, and cardiac function in PD patients [55]. Aaseth et al. (2018) have also suggested that by making appropriate lifestyle changes such as PE and intake of natural anti-oxidants help reduce deterioration of dopaminergic neurons, however, many other strategies are to be followed or compounds like iron binding agents and oxygen radical scavengers are also required [56]. Minakaki et al. (2019) reported improvement of gait activity, postural stability, and promotion of dopaminergic and α-synuclein homeostasis due to treadmill exercise in their study based on mice models of PD; however, no significant induction of cerebral ALP occurred due to it [57]. 

## 3. The Role of Exercise in Neurological Diseases and Involved Mechanisms

### 3.1. Neuroendocrine Regulation by Physical Exercise

PE is a stressor for the human body and acts as an activator of the neuro-endocrine system if the exercise is of sufficient intensity and/or duration [58]. Chronic exposure to exercise training leads to neuroendocrine system adaptations such as a decrease in hormonal stress response to sub-maximal exercise [58]. PE provokes many major changes in concentration of hormones like vasopressin, cortisol, β-endorphin, adreno-corticotropic hormone, and some others from resting levels. The greater the exercise volume (intensity and/or duration of exercise), the greater the neuroendocrine response [59]. PE begins a coordinated series of physiological responses that include the hypothalamic–pituitary–adrenal axis and sympathetic nervous system activation. This combination leads to the appropriate selection and use of metabolic substrates. It acts as a powerful stimulus for the hypothalamic–pituitary axis, but the nature of this stimulus depends on many factors like the kind of exercise (intensity, aerobic, duration, strength), time of the day, meal ingestion, and subject characteristics (previous training, gender) [60].

### 3.2. Neurotransmitter Regulation by PE

PE influences the central dopaminergic, seratonergic, and noradrenergic systems [61]. Peripheral physiological adaptations toward exercise occur to adjust to the disturbance in resting homeostasis, which is induced by exercise stimulus. Many experimental studies in which homogenized tissues have been used to check the level of different neurotransmitters indicate that changes in the synthesis and metabolism of monoamines and neurotransmitters occurs during exercise [61]. Application of micro-dialysis and voltammetry for measuring *in vivo* release neurotransmitters have indicated that then release of most neurotransmitters is influenced by exercise [61]. According to Lin and Kuo (2013), dopamine (DA), noradrenaline (NA), and serotonin or hydroxytryptamine(5-HT) are the three main monoamine neurotransmitters modulated by exercise [62] and their release is increased during exercise. The extracellular levels of dopamine, noradrenaline, serotonin,γ-amino butyric acid (GABA), and glutamate (GLU) are influenced by exercise training. Upregulation of DA in the brain is associated with exercise induced higher levels of serum calcium that is transported in the brain and effects calcium/calmodulin-dependent DA synthesis by activating the tyrosine hydroxylase enzyme [63]. In addition, the binding affinity between DA and its receptor, which is determined by [3H]spiroperidol binding, is enhanced by exercise training [64,65]. Furthermore, exercise provokes neuronal adaptation in response to uncontrollable stress [66]. This protective mechanism of PE against stress is due to the expression of galanin in the locus coeruleus [67], which hyperpolarizes noradrenergic neurons and inhibits neuronal firing by locus coeruleus, causing suppression of norepinephrine (NE) release [68]. NE, which targets the amygdala and frontal cortex, prohibits anxiety behavior upon decreased release [67]. NE also participates in the consolidation and retrieval of memory [69]. Chronic and wheel exercise both increase the levels of NE in spinal cord pons-medulla in comparison to sedentary controls [70] and the endogenous activity of NE is enhanced by exercise, showing a potential link between NE and exercise-enhanced cognitive function [62].

The HT system is modulated by exercise, but this modulation is dependent on the brain region and is also determined by the duration and intensity of exercise. For instance, four weeks of moderate treadmill exercise decreased the 5-HT levels with no effect on the metabolism of 5-HT in the hippocampus [71]. On the other hand, seven days of high-intensity treadmill exercise increased the levels of hippocampal 5-HT significantly [72].

### 3.3. Exercise-Enhanced Neural Insulin Signaling

Brain insulin signaling is required for neuronal survival and maintenance of crucial brain functions, and can both prevent and reverse the defects in the BDNF transport [73]. Insulin deregulation are connected with diabetes, obesity, cardiovascular disease, and hypertension and abnormal neural insulin signaling pathways are linked with various neurodegenerative diseases and learning memory deficits [74]. The insulin receptor (IR) is densely expressed in pyramidal cell axons in the hippocampal-CAl region and is mainly distributed in the dominant learning, memory, and cognitive function regions of the brain [75]. IR was found in different parts of the brain, however, it was mainly seen in the hypothalamus, hippocampus, and cerebral cortex at high concentrations [76]. Among these regions, the hippocampus plays an important role in storing new memories [77]. During normal physiological conditions, insulin and growth factors like BDNF, insulin like growth factors 1 and 2 (IGF-1 and 2) and VEGF transmit intracellular signals in the hippocampal neurons for their integrity and to keep hippocampus functional [78]. However, when the functionality of these growth factors is inhibited, the possibility of AD becomes high [78]. 

Aged rats showed decreased aversive memory as well as increased inflammatory markers such as TNFα, IL1-β, and NF-kβ, and decreased anti-inflammatory cytokine IL-4 and global histone H4 acetylation levels. However, forced exercise(running daily for 20 min for two weeks)reversed this effect in 20 month-old rats [79]. PE has been shown to exert anti-inflammatory effects and enhances insulin signaling in the hippocampal neurons [78]. PE also elicits an insulin sensitizing effect in the peripheral nervous system [80] and hence the possibility of it to bring about the same effect in the central nervous system and play a neuroprotective role is quite possible [81]. There have been many other experimental evidences to show that PE helps in neuroprotection through its effect on the peripheral nervous system as well as central nervous system. In the peripheral tissues, PE promotes uptake of glucose in an insulin independent way by activating protein kinase that is itself activated by AMP (AMPK) and mammalian target of rapamycin (mTOR) [82]. On the other hand, the central nervous system is affected by PE in quite different ways. For instance, it improves cognition and synaptic plasticity [83,84], along with increasing neurogenesis [85] and angiogenesis [86]. It is also shown to regulate the production and degradation of various neurotransmitters [87,88]. However, the complete understanding of the molecular mechanisms involved is still lacking. 

### 3.4. Exercise-Enhanced Brain-Derived Neurotrophic Factor BDNF-Signaling

**Figure 2:**
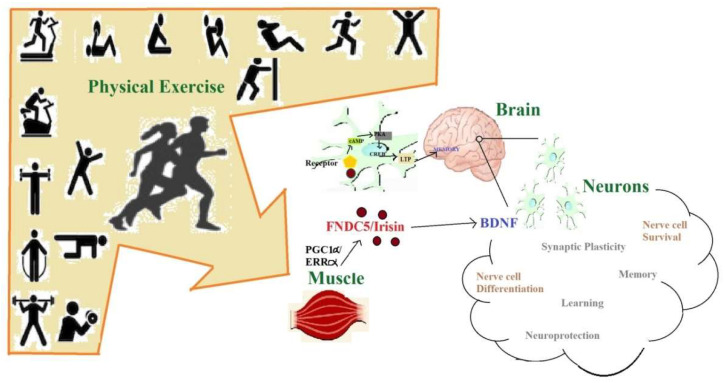
Effect of physical exercise (PE) on neurons involving brain-derived neurotrophic factor BDNF and irisin.

BDNF is a neurotrophin expressed in the hippocampus and is involved in processes related to memory and learning and is thought to play a crucial role in major depression [89]. BDNF is thus a central regulator of neuronal plasticity inside the post-natal hippocampus [89]. It plays an important role in synaptic plasticity and neuronal stress resistance [90]. It has an established role to play in promoting differentiation and maturation of developing neurons [91], while in mature neurons, it positively regulates the synaptic transmission and plasticity [92] and thus contributes to memory formation and learning [93]. Raichlen and Gordon (2011) reported a positive correlation between the size of the human brain and endurance exercise capacity, suggesting a co-evolution between locomotion and cognition in human [94]. Mattson et al. (2012) suggested that since endurance exercise clearly increases BDNF expression in the brain, improvement in exercise capacity may positively enforce brain growth, especially in hippocampus [95]. Considering the cases of peripheral nerve injury where transected fibers distal to the lesion are disconnected from the neuronal body, activity dependent therapy like early treadmill running decreases the synaptic stripping and disorganization of peri-neuronal nets on axotomized motor neurons. The underlying mechanisms that bring about such effects are not known, but the benefits of exercise are attributed to increase in BDNF [96]. Exercise training is known to enhance amygdala- and hippocampus-associated neuronal function [97]. Lin et al. (2015) also suggested that PE may serve as a way to delay the onset of Alzheimer’s disease on the basis of their APP/PS1 transgenic mice based study. It was reported that 10 weeks of treadmill training (from the age of 1.5 to four months) of the transgenic mice increased their dendritic arbor of CA1 and CA3 neurons, hippocampus-associated memory, restored the amygdala-associated memory, and dendritic arbor of amygdalar basolateral neurons [97]. Furthermore, they reported that PE increased the levels of BDNF/TrkB signaling molecules (p-AKT, p-PKC, and p-TrkB) in the hippocampus and amygdala, in addition to reducing the levels of soluble amyloid-β in both regions [97]. Fahimi et al. (2017) reported that around four weeks of treadmill and running wheel exercises in mice brought about many changes such as (1) significant increase in BDNF mRNA and protein levels; (2) significantly increased synaptic load in dentate gyrus; (3) changes in the morphology of astrocytes; and (4) orientation of astrocytic projections toward dentate gyrus cells [98]. These changes were possibly linked to an increase in TrkB receptor levels in the astrocytes [98]. Zsuga et al. (2016) suggested that BDNF modulates neuronal dopamine content and its release, which are essential for neuronal plasticity, neuronal survival, and learning and memory [99]. BDNF signaling is summarized in Figure 2.

### 3.5. Irisin Production and Secretion

Irisin is basically a myokine that is secreted from the muscles in response to exercise [100] in mice and human. FNDC5 is a muscle protein that is induced in exercise and is cleaved and secreted as irisin [101]. Irisin serves as a circulating myokine that increases thermogenesis and improves glucose homeostasis and obesity [102]. Wrann et al. (2013) found that forced expression of FNDC5 in primary cortical neurons increased expression of BDNF [101]. In addition, the peripheral delivery of FNDC5 to the liver through adenoviral vectors resulted in increased blood irisin, induced expression of BDNF, and other neuroprotective genes in the hippocampus [101]. Thus, through their study, Wrann et al. (2013) linked endurance exercise and metabolic mediators PGC-1α (regulator of neuronal Fndc5 gene expression) and FNDC5 (exercise induced) with BDNF expression in the brain [101]. Zsuga et al. (2016) also suggested on the basis of their study that irisin may be a link between physical activity and motivation and reward related processes [99] that are in turn related to the neurotransmitter dopamine, which itself gets activated through BDNF’s activity. Furthermore, Li et al. (2017) suggested that irisin decreases ischemia induced neuronal injury by activating Akt and ERK1/2 signaling pathways and thus contributes toward neuroprotective effects of exercise against cerebral ischemia [103]. This further indicates that irisin may be a factor that links metabolism and cardio-cerebrovascular disorders [103]. Another recent study by Peng et al. (2017) showed that irisin mitigates oxygen-glucose deprivation-induced neuronal injury in part by inhibiting the ROS-NLRP-3 (reactive oxygen species-Nod like receptor pyrin-3) inflammatory signaling pathway, indicating a possible mechanism for irisin induced therapeutic effects in ischemic stroke [104]. Another aspect of the beneficial effects of exercise is a reduction in neuropathic pain. Dameni et al. (2018) carried out their study in a chronic constriction injury model in male rats and found that acute administration of irisin increased pain threshold; however, irisin could not prevent the decline in the number of neurons [105]. Wang et al. (2018) reported on the basis of their study in primary cell cultures of astrocytes and neurons that a pretreatment of astrocyte-conditioned medium with irisin for about 12 h protected neurons from the toxicity of amyloid-β [106]. Irisin could also attenuate the release of IL-6 and IL-1β from cultured astrocytes and reduced expression of COX-2 and phosphorylation of AKT [106]. In addition, irisin could decrease NFκB activation of astrocytes exposed to amyloid-β by preventing phosphorylation and loss of IκBα [106]. Thus, irisin is supposed to be have a novel application in the treatment of AD and memory dysfunction in diabetes mellitus in the future [106]. 

### 3.6. Anti-Neural-Inflammatory and Anti-Neural-Oxidative Responses of PE

In response to PE, the autonomic nervous system and the hypothalamic–pituitary–adrenal axis come into action to maintain homeostasis. As a result, there is elevation in the level of cortisol and cathecholamines in plasma [107]. Exercise stimulates the secretion of growth hormone and prolactin and may influence the type of immunity by stimulating the TH2 response profile [107]. There have been attempts to identify potential biomarkers to characterize the response to exercise and to understand the molecular mechanisms leading to health benefits or mal adaptation due to physical activity, and such a study was conducted recently using 2D-gel electrophoresis followed by protein identification using liquid chromatography-tandem mass spectroscopy [108]. In this study done on six human subjects, it was found that 20 resolved serum proteoforms were significantly altered at 5 min and 1 h after high-intensity interval exercise, which included serpins (protease inhibitors), apolipoproteins, and immune system proteins that have broad antioxidant and anti-inflammatory effects and are involved in cardio-protective, neuro-protective effects, and lipid clearance [108]. There have been relevant studies to elucidate the synergistic effects of physical activity and anti-oxidants on neurons to act as a neuro-protective strategy in conditions like PD. One such study with a mice model of PD was recently done by Gil-Martinez et al. (2018), which involved the study of a combination of physical activity and an anti-oxidant named NAC (N-Acetyl-L-cysteine) as a neuro-protective strategy; however, this study reported that physical activity is beneficial, but in the long-term only and the combination of the NAC with physical activity brought about therapeutic benefits due to NAC only [109]. Another aspect of the effect of PE with reference to neuronal physiology is that PE produces intracellular as well as extracellular-heat shock proteins (iHSP70 and eHSP70, respectively) [110]. The activation of iHSP70 is an absolute requirement for promoting tissue repair, cyto-protection, and anti-inflammatory effect [110]. PE induces the appearance of HSP70 in extra cellular medium (eHSP70), which is involved in the activation of the immune system.Since, iHSP70 is unable to respond to stress in the motor neurons, the eHSP70 can be internalized by them to act as an intracellular chaperon, protecting the cell against protein denaturation and oxidative damage [110]. A lowered iHSP70 expression capacity is associated with neurodegenerative diseases like AD, PD, ALS, Huntington’s disease, and hence the elucidation of the role of eHSP70 can be helpful in treating these neurodegenerative disorders along with an understanding of the beneficial effects of PE in the neuronal cells [110]. Anti-oxidant enzymes like SOD (superoxide dismutase) become more active in response to exercise [30]. All these effects promote neuroplasticity, decrease neural apoptosis, and delay the neurodegeneration process, thus decreasing or preventing PD development [31].

### 3.7. Neural Pro-Survival and Anti-Apoptotsis Effects of PE

PE not only affects the activity of different brain cells, but also determines their survival and death. Recent evidence in a Long Evans rat model based study showed that voluntary exercise, in addition to enriched environment improves cognitive function, promoted neurogenesis and brain microvasculature in these rats exposed to hypobaric hypoxia at high altitudes by mediating VEGF signaling [111]. In a Wistar rat model, it was demonstrated that early PE in childhood and adolescence induces long term morphological alterations in hippocampal and cortical neurons even during the sedentary period of rats [112]. It is supposed that PE enhances the expression of neurotrophic factors and promotes neuronal growth, leading to usage of a neuronal reserve in later stages of life [112]. Furthermore, the study showed that exercise during juvenile stages increased and maintained the number of hippocampal and cortical neuronal cells and dendritic arborization [112]. In addition, the expression of survival proteins like cortical mTOR and hippocampal BDNF was found to be enhanced at P60, but were restored to control levels at P90 and P120 [112]. BDNF has been considered to be likely to also elicit the beneficial effects of exercise with regard to protection against dementia and type-II diabetes [113]. Another study done in rat models showed that changes in the expression of inflammatory and cell survival proteins in the brain region of aged rats depended on the type of PE training [114]. The aerobic training increased expression of proteins such as p38, Akt, ERK, and p70S6k in the cortex of the brain [114]. Another recent study based on middle aged APP/PS1 transgenic mice with AD showed the protection of neurons and adult neurogenesis in the dentate gyrus and thus showed improved memory and spatial learning due to running exercise [115].

In aged PS2 mutant mice, treadmill exercise prevented PS2 mutation-induced memory impairment and decreased Aβ-42 deposition by inhibiting β-secretase (BACE-1) and its product C-99 in the hippocampus and/or cortex of these mice [116]. In the same study, it was found that treadmill exercise downregulated expression of GRP78/Bip and PDI proteins along with inhibiting the activation of PERK, ATF6α, eIF2α, sXBP1, and JNK-p38 MAPK [116]. Furthermore, it activates caspase-3, -12, and CHOP; upregulates expression of Bcl-2; and downregulates Bax expression in the hippocampus of aged PS2 mutant mice [116]. Varying intensities of PE have different effects on the nervous system, for example, instead of high intensity exercise, moderate intensity treadmill exercise has a neuroprotective effect in rats suffering from cerebral ischemia. Thus, it is speculated that due to high intensity treadmill exercise, the neurotrophic factors were downregulated, further affecting the expressions of cell cycle-related proteins [117].

Voluntary running is considered a powerful neurogenic stimulus that triggers proliferation of progenitor cells in the dentate gyrus, which is the site for adult neurogenesis occurring throughout life [118]. The retinal ganglion cells that become increasingly vulnerable to injury with aging also become protected through exercise, which is because PE prevents the loss of BDNF in retina post-injury, along with preserving neuronal function and survival by the prevention of complement mediated elimination of the synapses [119]. The examination of the effects of different intensities of aerobic exercise on resting serum BDNF, IGF-1 concentration, and cortisol, the hormone released in response to stress and memory of adolescent human, has been done in order to understand how PE brings changes in their expression [120]. For this, 40 adolescent males were recruited who performed aerobic exercise of moderate to high intensity, and it was found that PE also had a positive effect on the serum levels of BDNF at rest and on cognitive function [120]. 

### 3.8. Autophagy

Evolution favored organisms with superior physical and cognitive abilities under stressful conditions like limited food sources, and hence the brain function can be optimized by intermittent dietary energy restriction and exercise [31]. These energy challenges engage various cellular stress-response signaling pathways in the neurons involving protein chaperones, neurotrophic factors, DNA-repair proteins, mitochondrial biogenesis, and autophagy [31]. Lack of physical activity, overeating, and suppressing adaptive cellular stress responses thus may increase the risk of AD, PD, depression, and stroke [31]. Mattson (2014) suggested that interventions like exercise intermittent energy restriction can counteract neurodegenerative processes and improve brain function in animal models. This is because these interventions may support neuronal adaptive stress response pathways that enhance DNA repair, neurotrophic signaling, mitochondrial biogenesis, and proteostasis [121]. Pathways involving Ca^2+^, CREB, NFκB, and PGC-1α are activated in neurons upon physical activity (aerobic exercise) and food deprivation and these stimulate cellular stress response and mitochondrial biogenesis [122]. 

Autophagy ensures lysosome mediated breakdown and self-material recycling as it degrades damaged intracellular components and provides building blocks for biosynthetic and energy production [123]. Many animal model based studies, along with those involving drosophila, have shown that defects in the autophagic process cause a rapid decline in neuro-muscular function, sensitivity to stress conditions like starvation or oxidative damage, neurodegeneration, and stem cell loss [123]. Impairment of the autophagic pathway is known to play a role in β-amyloid production and AD progression following a complex mechanism. In the Alzheimer’s disease mice model based study, it was found that when autophagy is genetically hyperactivated by knocking-ina gene-point mutation (Becn1^F121A^) in the autophagy essential gene (Beclin 1/Becn 1), a significant decrease in amyloid accumulation is observed and there is a prevention of cognitive decline along with restoring the survival of AD mice.This happens because the F121A point mutation induced in Becn 1 significantly decreases the interaction of BECN 1 with its inhibitor BCL2, leading to constitutively active autophagy even in non-autophagy inducing conditions [124]. It was observed that biochemically, amyloid-β-oligomers are autophagic substrates and sequestered inside autophagosomes in the brain of autophagy hyperactive AD mice [124]. The same study suggested that voluntary exercise is a physiological autophagy inducer and exerts similar Becn1-dependent protective effects on amyloidβ removal and memory in these AD mice [124].

## 4. Conclusions

Physical activities have been proven to have beneficial effects on the general health and well-being of the people who exercise on a regular basis. Each and every part of the body is benefitted in one way or another among regular exercisers. Talking specifically about their effects on neuronal cells and brain function, there are many research-based evidences that prove that PE has neuroprotective effects. Physical activities elicit their benefits through some signaling mechanisms that have, however, not been completely elucidated to date, but neurotrophins like BDNF, hormones like irisin, and neurotransmitters like dopamine are direct participants in these mechanisms. Considering its effect among PD patients, it improves gait, balance, cognition, along with a slowing down progression of the disease by avoiding protein aggregation in the brain. In AD patients, it also slows down the progression of the disease, along with improvement in cognition, memory, and delays the onset of neuro-psychiatric symptoms like depression, apathy, and others. The different physiological aspects affected by PE are:hippocampal insulin signaling, autophagy, anti-oxidant and anti-inflammatory responses, cell survival and death pathways. Physical activities enhance the expression of BDNF, which is an essential mechanistic step involved in the beneficial process occurring due to them. Molecules like dopamine, irisin, GABA, and Aktare also involved in these mechanisms. Still, PE cannot be applied as a stand-alone way to handle neuro-pathologies. As an add-on therapy; however, it has great potential in this regard.
